# Improved Black-Winged Kite Algorithm with Multi-Strategy Optimization for Identifying *Dendrobium huoshanense*

**DOI:** 10.3390/biomimetics10040226

**Published:** 2025-04-04

**Authors:** Chaochuan Jia, Ting Yang, Maosheng Fu, Yu Liu, Xiancun Zhou, Zhendong Huang, Fang Wang, Wenxia Li

**Affiliations:** 1 College of Electronics and Information Engineering, West Anhui University, Lu’an 237012, China; 03000076@wxc.edu.cn (C.J.);; 2 Anhui Province Intelligent Hydraulic Machinery Joint Construction Subject Key Laboratory, Lu’an 237012, China; 3 Anhui Dabieshan Academy of Traditional Chinese Medicine, West Anhui University, Lu’an 237012, China; 4 College of Electrical and Optoelectronic Engineering, West Anhui University, Lu’an 237012, China

**Keywords:** black-winged kite optimization algorithm, opposition-based learning, differential mutation, random boundary, identified *Dendrobium huoshanense*

## Abstract

An improved black-winged kite algorithm with multiple strategies (BKAIM) is proposed in this paper to address two critical limitations in the original black-winged kite optimization algorithm (BKA): the restricted search capability caused by the low-quality initial population and the reduced population diversity resulting from blind following behavior during the migration phase. Our enhancement implements three strategic modifications across different algorithm stages. During initialization, an opposition-based learning strategy was incorporated to generate a higher-quality initial population. For the migration phase, a differential mutation strategy was integrated to facilitate information exchange among population members, mitigate the tendency of blind leader-following behavior, enhance convergence precision, and achieve an optimal balance between exploration and exploitation capabilities. Regarding boundary handling, the conventional absorption boundary method was replaced with a random boundary approach to increase population diversity and subsequently improve the algorithm’s search capabilities. Comprehensive testing was conducted on four benchmark function sets (CEC2017, CEC2019, CEC2021, and CEC2022) to validate the effectiveness of the improved algorithm. Detailed convergence analysis and Wilcoxon rank-sum test comparisons with other algorithms demonstrated BKAIM’s superior convergence performance and robustness. Furthermore, the support vector machine (SVM) model was optimized by BKAIM for grade identification of *Dendrobium huoshanense* based on near-infrared spectral data, thereby confirming its effectiveness in practical applications.

## 1. Introduction

Over the past decade, optimization problems in the engineering field are showing an increasingly complex trend. To address the issue that traditional optimization algorithms cannot efficiently solve complex optimization problems, swarm intelligence optimization algorithms with simple structures and high efficiency have emerged, and they have found extensive applications across various domains, including feature selection [1], pattern recognition [2], signal processing [3], path planning [4], wireless sensor networks [5], power forecasting [6], and so on. Recently, numerous scholars have drawn upon the predatory behaviors and living habits of organisms in nature to innovatively propose a series of swarm intelligence optimization algorithms, injecting new vitality into the field of intelligent computing, such as the GOOSE algorithm [7], artemisinin optimization (AO) [8], fox optimization algorithm (FOX) [9], Harris Hawks optimization (HHO) [10], whale optimization algorithm (WOA) [11], Beluga whale optimization (BWO) [12], and so on. 

Introducing a novel approach in swarm intelligence, the black kite optimization algorithm (BKA), proposed in 2024, distinctively emulates the dual behavioral patterns—predatory attacks and migratory movements—exhibited by black kites, setting it apart from conventional swarm-based optimization methods. It has a small number of parameters and is simple to operate due to its dual-mode attack behavior and the structure of the dynamic leadership mechanism, and it has excellent global optimization ability and adaptability [13]. Despite its innovative design, the black kite optimization algorithm (BKA) shares common limitations inherent to swarm intelligence (SI) optimization algorithms, specifically regarding inadequate convergence accuracy and a suboptimal convergence rate.

Currently, there are few improved works on BKA. Nevertheless, a significant body of research has emerged, as scholars have developed diverse enhancement methodologies to overcome the inherent limitations of swarm intelligence optimization algorithms [14]. These improved strategies can be roughly categorized into four mainstream directions: (1) population initialization optimization, targeting improved diversity and quality of initial solutions, (2) individual position update refinement, facilitating better balance between exploratory and exploitative phases while minimizing optimization errors, (3) hybrid algorithm construction, strategically combining complementary strengths from multiple optimization approaches, and (4) boundary processing improvement, ensuring sustained population diversity throughout the optimization process.

In the initial population generation phase of most swarm intelligence optimization algorithms, individuals are typically created randomly within a prescribed range, leading to significant randomness and uncertainty in the initial population. To address this, chaotic mapping was incorporated during population initialization to facilitate a more comprehensive traversal of the search space, resulting in a more uniformly distributed initial population [15,16,17]. Additionally, opposition-based learning was employed to generate antithetical positions of the initial population. Utilizing a greedy strategy, individuals with smaller fitness values were selected to enhance the quality of the initial population, which contributes to accelerated convergence [18,19,20].

In terms of optimizing the individual position update method, numerous improvement strategies have been proposed. Taher et al. implemented a mutation-update strategy within the grasshopper optimization algorithm, effectively expanding its exploration capabilities and improving its resilience against getting trapped in local optima [21]. Zhou et al. integrated phasor and flow-direction operators into the eagle optimization algorithm to enhance its optimization performance in high-dimensional spaces [22]. Ahmed et al. enhanced the genetic algorithm by adopting the direction-crossing and normal mutation strategies, enabling it to perform excellently in both low-dimensional and high-dimensional cases, significantly enhancing the convergence rate and exploration capability of the genetic algorithm [23]. The differential evolution strategy was employed by Binanda et al. to update individuals in the crayfish optimization algorithm, which effectively enhanced the global optimization capability of the algorithm. Remarkable performance was demonstrated in both practical engineering applications and benchmark testing functions [24]. The chimp optimization algorithm was enhanced by Li et al. through the introduction of chaotic initialization and Cauchy mutation, which significantly increased the diversity and uniform distribution of the initial population while improving the global exploration capability. The improved algorithm demonstrated outstanding performance in both natural image and medical image segmentation [25]. The performance of the slim mold algorithm was enhanced by Gauri et al. through the introduction of the Gaussian mutation strategy. The improved algorithm was demonstrated to surpass the original algorithm in the areas of stability, convergence speed, and reliability [26].

Within hybrid optimization algorithm research, by comprehensively leveraging the differences and complementarities of multiple optimization algorithms, it is possible to achieve complementary advantages among algorithms. As a result, the simulated annealing algorithm’s formulation was embedded into the whale optimization algorithm by Mafarja et al., with the goal of amplifying its global exploration efficiency and elevating its accuracy in optimization [27]. The grasshopper optimization algorithm and the Harris Hawks optimization algorithm were integrated into a hybrid optimization algorithm by Murugan et al., which was found to exhibit exceptional performance when applied to the combined heat and power economic dispatch problem [28]. A hybrid intelligent optimization algorithm was developed by Deng et al. through the combination of the benefits of multiple algorithms, including improved particle swarm optimization, differential evolution, simulated annealing, and quantum-behaved particle swarm optimization, aimed at enhancing global search efficiency and stability [29]. By integrating the advantages of the particle swarm optimization algorithm and the genetic algorithm, a hybrid algorithm was proposed to optimize the performance of the mobile edge computing monitoring network for non-orthogonal multiple access [30]. Similarly, practical engineering problems have been tackled through the proposal of hybrid algorithms, such as the combination of the differential evolution and ant colony optimization algorithms [31], and the merging of the GA, DE, and PSO algorithms [32].

In terms of the boundary handling method, which effectively utilizes boundary information to adjust the search strategy, it enhances the even dispersion of the population, prevents excessive concentration of individuals, and avoids invalid searches. For instance, the problem of individual out-of-bounds situations was addressed by Wu et al. through the application of the double-mirror reflection theory, which resolved the issue of uneven population distribution and consequently enhanced the optimization capability of the golden jackal optimization algorithm [33]. By employing the law of reflection, Cheng et al. effectively addressed the treatment of individuals, leading to an enhancement in the search ability of boundary individuals [34]. An adaptive boundary strategy was developed by Feng et al. in the whale optimization algorithm, resulting in an improvement in its solution efficiency and quality [35]. The whale optimization algorithm’s population diversity and stability were maintained by Yu et al. by employing a collision-rebound mechanism to address out-of-bounds situations [36]. A contraction-processing method in a specific direction was proposed by Xuan et al. to address individual out-of-bounds situations, thereby enhancing the performance of the grey wolf optimization algorithm [37]. By employing the principle of convex-lens imaging, Huang et al. mapped out-of-bound individuals back to the feasible solution space, which not only preserved the population diversity but also significantly boosted the search performance of the artificial rabbit optimization algorithm [38].

Although various enhancement strategies have been developed, their impacts can vary significantly across different optimization algorithms. There is no universal improvement strategy that is applicable to all optimization algorithms. Consequently, for a specific optimization algorithm, it is essential to carefully evaluate and integrate multiple improvement strategies to achieve optimal results.

The BKA achieves a balance between exploration and exploitation during the attack phase by incorporating two modes: high-altitude diving and short-distance precise attacks. While this improves the algorithm’s global search capability and reduces the likelihood of local optima convergence, its search efficiency is hindered by the lack of information exchange among individuals during this phase. In the migration phase, the BKA adopts a dynamic leader selection mechanism, enabling the population to adaptively change leaders based on fitness values and guiding them toward the optimal solution. However, the tendency for all individuals to blindly follow the optimal leader during migration results in reduced population diversity. To overcome these challenges, an improved black kite optimization algorithm is proposed in this paper, which integrates multiple strategies for enhanced performance.

The principal contributions of this paper are summarized as follows:

- During initialization, an opposition-based learning strategy was incorporated to generate a higher-quality initial population.
- For the migration phase, a differential mutation strategy was integrated to facilitate information exchange among population members, mitigate the tendency of blind leader-following behavior, enhance convergence precision, and achieve an optimal balance between exploration and exploitation capabilities.
- Regarding boundary handling, the conventional absorption boundary method was replaced by a random boundary approach to increase population diversity and subsequently improve the algorithm’s search capabilities.

The remaining sections of this paper are organized as follows: an introduction of the BKA is provided in Section 2, Section 3 details the three improvement strategies, Section 4 presents the experimental analysis, and Section 5 provides the conclusions.

## 2. Black-Winged Kite Optimization

The BKA, which draws inspiration from the hunting and migratory behaviors of the black-winged kite, is a novel metaheuristic optimization algorithm introduced by Wang Jun et al. in 2024 [13]. This algorithm is composed of three distinct phases: the initialization stage, the attacking stage, and the migratory stage.

### 2.1. Initialization Stage

Similar to other SI optimization algorithms, a random initialization method is employed by the black-winged kite optimization algorithm to generate the initial population within the defined upper and lower bounds of the search range. This process can be mathematically expressed as:

(1) $$X_{i}=LB+rand\times (UB-LB)$$

where $X_{i}$ represents the *i*th individual, rand is a randomly generated number within the range of 0 to 1, and UB and LB denote the upper and lower bounds of the search range, respectively.

### 2.2. Attacking Stage

At this stage, the black-winged kite exhibits its unique hunting behavior by hovering in mid-air to locate prey. It achieves this by dynamically adjusting its wing and tail angles in response to wind speed, followed by a swift dive to capture the target. This stage incorporates two distinct attacking strategies by the constant p. The process can be mathematically modeled as:

(2) $$X_{i}(t+1)=\left\{\begin{array}{ll} X_{i}(t)+n(1+\sin(r))\times X_{i}(t) & if\;p<r\\ X_{i}(t)+n(2r-1)\times X_{i}(t) & else \end{array}\right.$$

(3) $$n=0.05\times \exp(-2\times {\left(t/T\right)}^{2})$$

In the above formula, the first equation models the black-winged kite’s hovering behavior, where it spreads its wings and maintains balance while actively scanning for prey. The second equation represents the black-winged kite’s rapid descent at an exceptionally high velocity to attack the prey. $X_{i}(t)$ and $X_{i}(t+1)$ represent the positions of the *i*th black-winged kite in the *t*th iteration and the (*t* + 1)th iteration, respectively, r is the random number within the range of [0, 1], p equals 0.9, sin(•) is the sine function,exp(•) represents the exponential function, t is the current iteration, and T is the maximum iteration.

### 2.3. Migratory Stage

The migration phase of the BKA simulates the natural leadership dynamics observed in bird flock migration. This mechanism employs a fitness-based evaluation to determine leadership roles during the collective movement process. Specifically, the algorithm compares the fitness values between the current individual and a randomly selected counterpart to assess leadership suitability. When the current individual demonstrates inferior fitness compared to its randomly selected counterpart, it indicates inadequate leadership capabilities, resulting in its transition from a leadership role to part of the following population. Conversely, when the current individual maintains superior fitness, it continues to serve as the migration leader, guiding the population toward the destination. This fitness-driven leadership selection mechanism ensures adaptive, performance-based guidance throughout the migration process, optimizing the collective movement strategy. The mathematical formulation of this migration phase can be expressed as follows:

(4) $$X_{i}(t+1)=\left\{\begin{array}{ll} X_{i}(t)+C(0,1)\times (X_{i}(t)-X_{L}(t)) & if\;F_{i}<F_{r}\\ X_{i}(t)+C(0,1)\times (X_{L}(t)-m\times X_{i}(t)) & else \end{array}\right.$$

(5) m=2×sin(r+π2)

(6) C(0,1)=tan(rand(1,dim)−0.5)×π)

where $X_{L}(t)$ is the optimal solution in the population at the *t*th iteration and is also the leader of the current population. $F_{i}$ denotes the fitness value of the *i*th individual, $F_{r}$ is the fitness value of any other individual, C(0,1) denotes the Cauchy mutation coefficient, dim represents the size of the dimension, r is the random number within the range of [0, 1], sin(•) is the sine function, rand(•) is the function to generate a random matrix with values uniformly distributed between 0 and 1, and tan(•) denotes the tangent function.

## 3. Improved Black-Winged Kite Optimization

The BKA faces two significant challenges: a tendency to prematurely converge to local optima and a suboptimal convergence speed. To address these limitations, this study implemented a comprehensive enhancement strategy across multiple phases of the algorithm. During population initialization, the traditional random approach was augmented with an opposition-based learning mechanism, which systematically generated complementary solutions within the search space. These opposition solutions were then rigorously evaluated against their original counterparts using a greedy selection strategy, ensuring the initial population comprised high-quality, well-distributed solutions.

In the migration phase, where the algorithm’s traditional leader-following mechanism often leads to premature convergence, a differential mutation strategy was introduced. This approach leveraged the informational differences between population members to guide the search process toward more promising regions of the solution space, effectively mitigating the risk of stagnation in local optima. Furthermore, the boundary handling mechanism was refined by replacing the conventional absorbing boundary method with a stochastic approach. This modification enhanced population diversity by allowing controlled exploration beyond the defined boundaries, thereby improving the algorithm’s ability to thoroughly investigate the entire solution space and escape from local optima traps.

### 3.1. Opposition-Based Elite Initialization Method

Traditional population initialization in the BKA relies on random generation, which often results in suboptimal distribution of individuals within the search space, characterized by inherent randomness and limited coverage. This limitation significantly impacts the algorithm’s optimization efficiency during initial phases. To address this challenge, this study incorporated an opposition-based learning (OBL) strategy, originally developed by Tizhoosh et al. in 2005, into the population initialization framework. The OBL approach systematically generates complementary solutions that are positioned opposite to randomly generated individuals within the search space.

This methodology enhanced the global search capability through two primary mechanisms: first, by effectively expanding the exploration space beyond the conventional random initialization and, second, by providing a structured method for generating diverse solutions that explicitly consider symmetrical positions relative to existing points. The implementation process evaluated both the original and opposition solutions through fitness comparisons, selectively retaining the superior solution for each individual. This dual-phase initialization process not only improved the quality of the initial population but also established a more informed starting point for subsequent optimization processes. The position-updating mechanism integrating this opposition-based learning strategy can be modeled as follows:

(7) $$X_{i}^{\prime }=UB+LB-X_{i}$$

(8) $$X_{i}=\left\{\begin{array}{ll} X_{i}^{\prime } & if\;f(X_{i}^{\prime })<f(X_{i})\\ X_{i} & else \end{array}\right.$$

where $X_{i}$ is the current individual, $X_{i}^{\prime }\;$ is the opposite position of $X_{i}$, and f(•) is the fitness function, while UB and LB denote the upper and lower bounds of the search range, respectively.

### 3.2. Differential Mutation Strategy

The migration phase of the BKA currently positions all individuals to follow the population leader, which presents a significant limitation when the leader becomes trapped in local optima, causing the entire population to stagnate. To address this critical issue, this study introduced a differential mutation strategy that enhances the algorithm’s exploratory capabilities and reduces the population’s over-reliance on the leader. The proposed approach systematically applied mutation operations to each individual’s current position, generating new potential solutions. These mutated solutions were then rigorously evaluated against their original counterparts through fitness comparisons, with the superior solution retained in the population. This mechanism effectively balances exploration and exploitation by maintaining beneficial diversity while preserving successful solutions. The strategy’s implementation not only improved the algorithm’s ability to escape local optima but also enhanced its global search capabilities. The position-updating process incorporating this differential mutation strategy can be mathematically formulated as follows:

(9) $$X_{i}^{\prime }=X_{i}+r_{1}*(X_{best}-X_{i})+r_{2}*(X_{j}-X_{k})$$

(10) $$X_{i}=\left\{\begin{array}{ll} X_{i}^{\prime } & if\;f(X_{i}^{\prime })<f(X_{i})\\ X_{i} & else \end{array}\right.$$

where $X_{best}$ represents the optimal solution within the current population, $X_{j}$ and $X_{k}$ are two random solutions that are different from $X_{i}$, $X_{i}^{\prime }$ represents the new solution in the current iteration, $r_{1}$ and $r_{2}$ are the random numbers within the range of [0, 1], and f(•) is the fitness function.

### 3.3. Stochastic Boundary Method

The BKA traditionally employs the absorbing boundary method to handle individuals that exceed the search space boundaries, which involves repositioning them directly onto the boundary. While this approach ensures feasibility, it inherently reduces population diversity and may lead to premature convergence, potentially causing the algorithm to miss the global optimal solution. To overcome this limitation, this study introduced the stochastic boundary method as an alternative boundary handling strategy.

**Figure 1:**
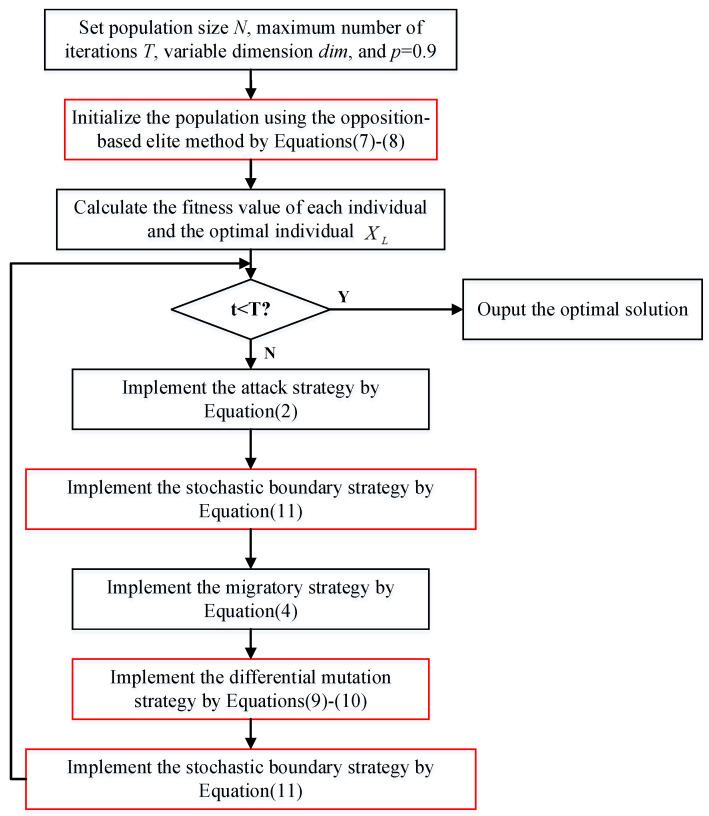
The process of the improved BKA.

The stochastic boundary method offers significant improvements by maintaining population diversity while ensuring feasible solutions. This method simulates more natural behavior, where individuals probabilistically rebound within the search space rather than being strictly confined to the boundary. The approach demonstrates enhanced adaptability, particularly in optimization problems with complex geometric boundaries or dynamically changing constraints. These characteristics make the method more aligned with real-world physical phenomena and improve the algorithm’s overall search performance. The position-updating mechanism for individuals can be mathematically modeled as follows:

(11) $$X_{i,j}=Lb_{j}+r_{3}*(Ub_{j}-Lb_{j})if\;X_{i,j}>Ub_{j}||X_{i,j}<Lb_{j}$$

where $X_{i,j}$ is the position of the *j*th dimension of the *i*th individual, $Lb_{j}$ and $Ub_{j}$ are the lower bound and the upper bound of the *j*th dimension, respectively, and $r_{3}$ is the random numbers within the range of [0, 1]. The process of the improved BKA is shown in Figure 1.

## 4. Simulation Experiments and Results Analysis

To comprehensively evaluate the enhanced BKA’s efficacy, a dual-pronged experimental approach was implemented, encompassing both benchmark function optimization and practical engineering problem-solving scenarios. The comparative analysis included seven state-of-the-art algorithms: BKA, WOA, GOOS, A, FO, HHO, and BWO, as competing methods.

The experimental framework employed standard benchmark suites from CEC2017, CEC2019, CEC2021, and CEC2022 to rigorously assess algorithmic performance. To mitigate the inherent stochastic characteristics of metaheuristic algorithms and ensure statistical reliability, each experimental configuration was independently executed 30 times, with performance metrics computed as arithmetic means. The experimental parameters were standardized across all algorithms: maximum iteration count = 300, population size = 30, and dimensionality = 10, ensuring fair comparison conditions.

### 4.1. Experiments on Four Benchmark Function Sets

**Figure 2:**
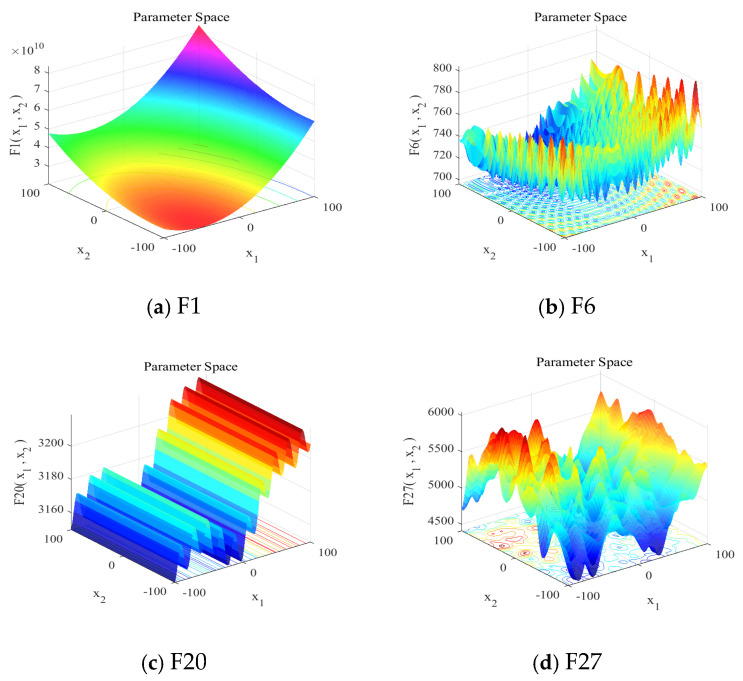
The 3D surface plots of selected benchmark functions on CEC2017: (**a**) F1, (**b**) F6, (**c**) F20, and (**d**) F27.

To conduct a comprehensive performance evaluation, we utilized an extensive set of benchmark functions, comprising 29 from CEC2017, 10 from CEC2019, 10 from CEC2021, and 12 from CEC2022. All test functions were configured with 10-dimensional search spaces bounded within [−100, 100], targeting minimization objectives. Figure 2 presents graphical representations of the selected CEC2017 test functions, with detailed specifications available in the corresponding reference literature.

**Figure 3:**
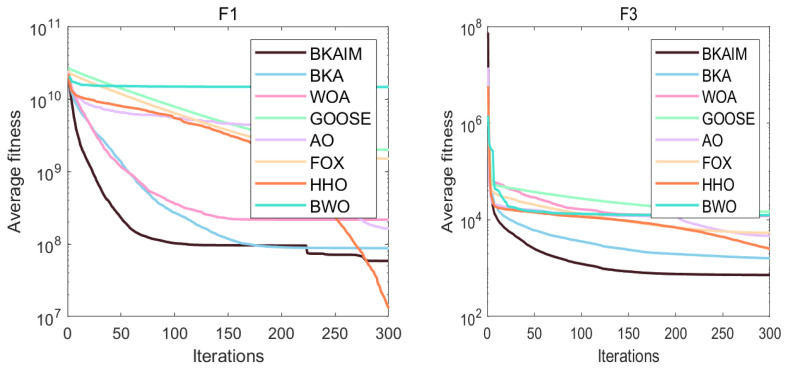
The average convergence curves of unimodal functions on CEC2017.

**Figure 4:**
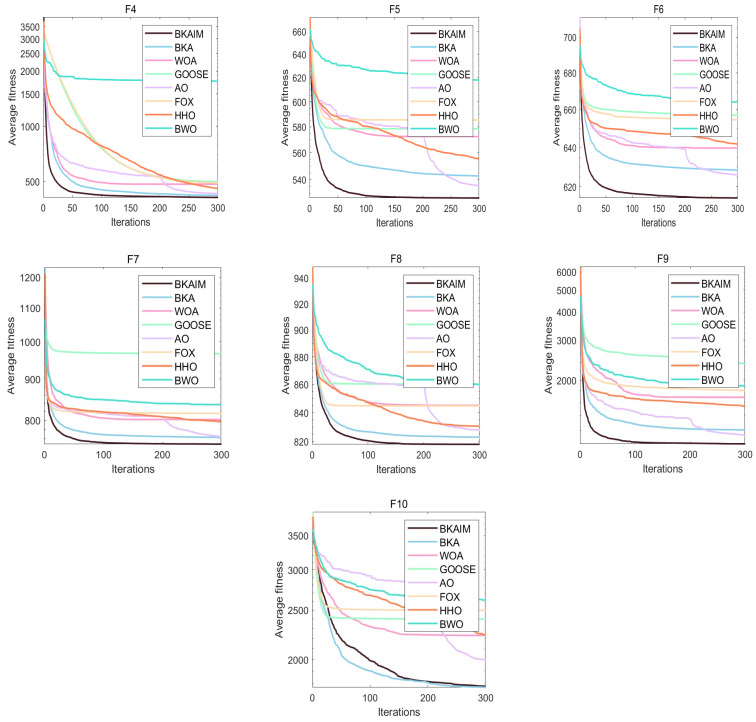
The average convergence curves of multimodal functions on CEC2017.

**Figure 5:**
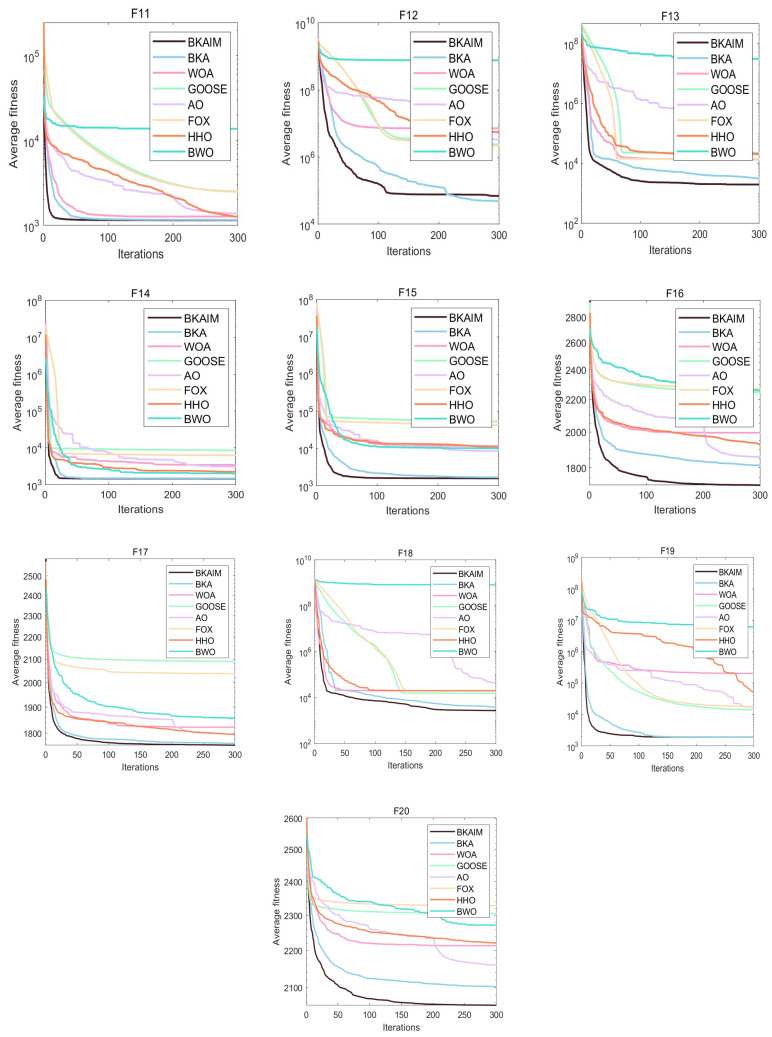
The average convergence curves of hybrid functions on CEC2017.

**Figure 6:**
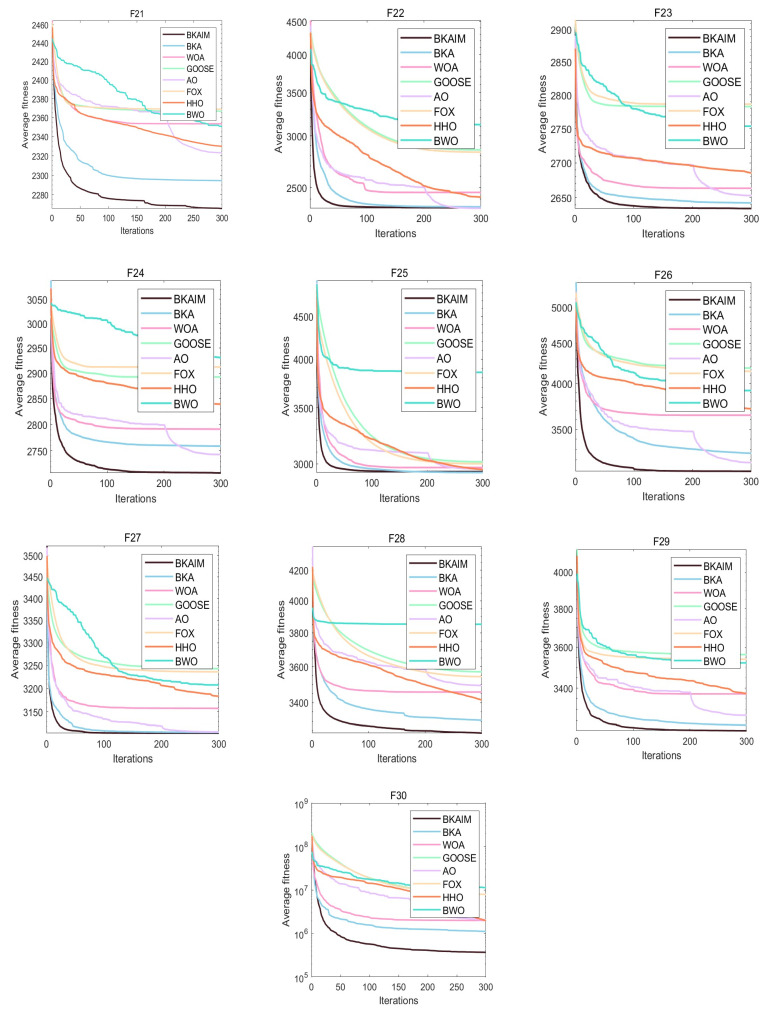
The average convergence curves of composition functions on CEC2017.

Addressing the inherent stochastic nature of optimization algorithms, each algorithm was independently executed 30 times under identical conditions to ensure statistical reliability and facilitate robust performance analysis. The experimental results are visualized through average convergence curves in Figure 3, Figure 4, Figure 5 and Figure 6, accompanied by comprehensive statistical metrics (including optimal value, standard deviation, and mean value) documented in Table 1. From Figure 3, it can be observed that for unimodal functions, BKAIM outperformed other algorithms in all cases except F1 (where it was slightly inferior to HHO) and achieved optimal convergence and accuracy on F3. From Figure 4, for multimodal functions, BKAIM demonstrated superior convergence and accuracy on F4 through F9, though it was slightly outperformed by BWO on F10. From Figure 5, for hybrid functions, BKAIM achieved the best convergence and accuracy on F11 and F13 through F20, with the exception of F12, where it was slightly inferior to BKA. From Figure 6, for composition functions, BKAIM exhibited optimal convergence and accuracy on F21 and F23 through F30, excluding F22, where it was slightly surpassed by AO. These findings are further supported by Table 1, where the best average values are highlighted in bold.

The statistical analysis demonstrated significant performance improvements of the BKAIM over its original version across the majority of test functions. Furthermore, the modified algorithm displayed enhanced exploration and exploitation capabilities, enabling more accurate and rapid identification of optimal solutions. These performance advantages of the enhanced BKA were consistently observed across all evaluated test suites (CEC2017, CEC2019, CEC2021, and CEC2022), thereby validating the effectiveness of the proposed algorithmic improvements.

**Figure 7:**
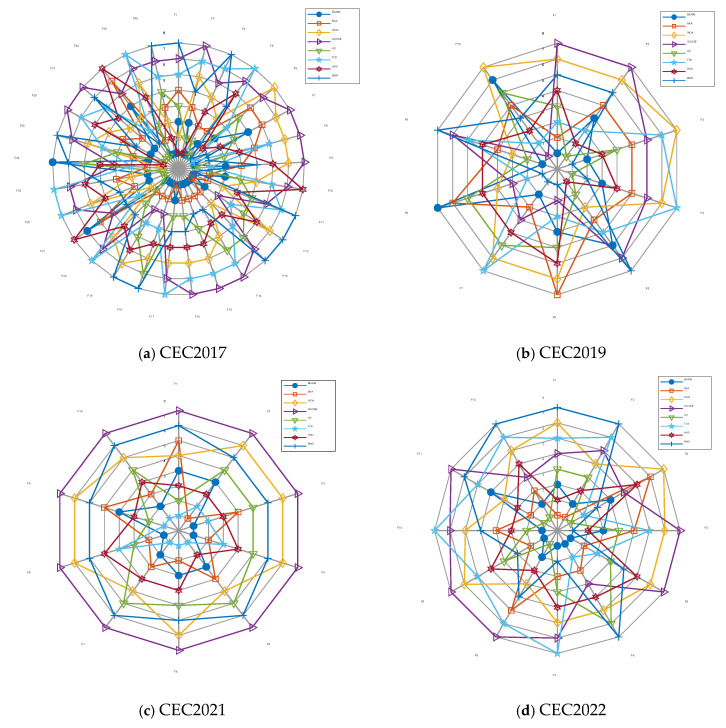
Radar chart of optimization results on four benchmark function sets.

**Figure 8:**
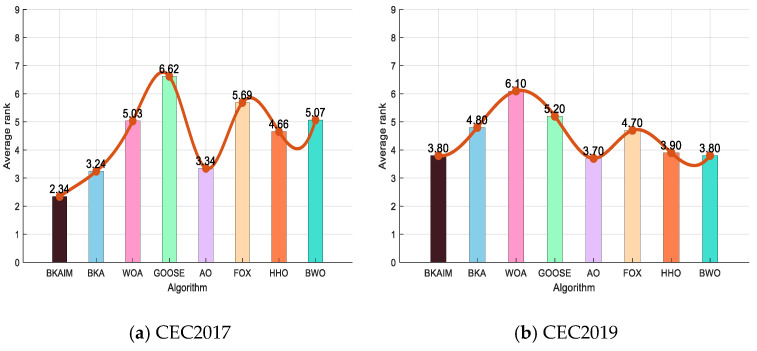

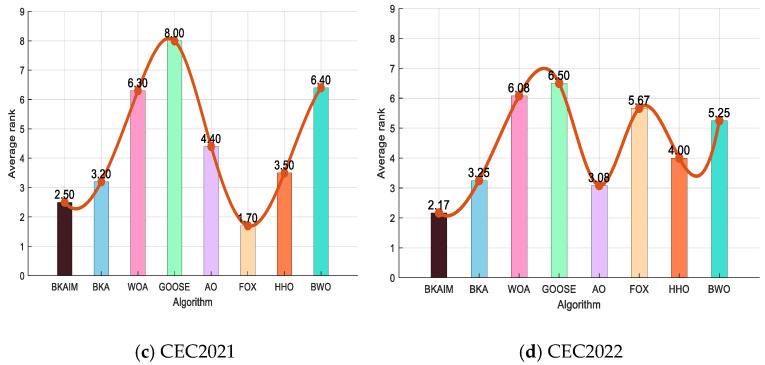
The results of the average rank on four benchmark function sets.

To comprehensively evaluate the improved BKA’s performance, we conducted comparative experiments encompassing the improved BKA, its original version, and six prominent swarm intelligence algorithms across three established benchmark suites: CEC2019, CEC2021, and CEC2022. While the detailed experimental results, including average convergence trajectories, box plot visualizations, and comprehensive statistical tables from these benchmarks, could not be included due to space constraints, the critical comparative analyses have been preserved. Specifically, the algorithm performance has been illustrated through multidimensional radar charts and integrated ranking diagrams, as systematically presented in Figure 7 and Figure 8, respectively. In Figure 7, the radar chart includes axis labels along each radiating signal line, representing the average values obtained by optimization algorithms, and the center point denotes 0, with values increasing outward along each axis. Each directional axis corresponds to a different function.

The comparative performance analysis presented in Figure 7 reveal notable superiority of the improved BKA over the baseline BKA across multiple benchmark testbeds. Specifically, the improved algorithm demonstrated superior performance on 26, 7, 10, and 9 functions from the CEC2017, CEC2019, CEC2021, and CEC2022 test suites, respectively. These empirical results substantiate that the algorithmic enhancements implemented in BKA contribute to significantly improved optimization capabilities across diverse problem domains.

The experimental results depicted in Figure 8 demonstrate the superior performance metrics of the improved BKA across all four benchmark functions. Specifically, the improved version achieved first-place rankings among the eight evaluated algorithms on both CEC2017 and CEC2022 test suites, while securing second-place positions on CEC2019 and CEC2021. These consistent competitive outcomes not only validate the exceptional search and optimization capabilities of the modified algorithm but also empirically substantiate the effectiveness of the proposed enhancements in the BKA framework.

### 4.2. Nonparametric Test Analysis

While the previous simulation experiments demonstrated the superiority of the enhanced BKA, concerns regarding result reproducibility and statistical significance necessitated further validation. To rigorously evaluate performance differences between the improved BKA, original BKA, and competing algorithms, comprehensive nonparametric statistical analyses were conducted on the CEC2017 benchmark functions. The Wilcoxon rank-sum test, selected for its distribution-free properties and robustness against parametric assumptions, was systematically employed across all comparative evaluations. Statistical significance was established at a threshold of *p* < 0.05, with values exceeding this threshold highlighted in bold within Table 2 for clarity.

The statistical analysis revealed nuanced performance characteristics: across ten test functions, the improved BKA exhibited insignificant differences (*p* > 0.05) from its original counterpart, a finding consistent with the theoretical framework, wherein the algorithm’s core architecture remained intact while incorporating three strategic enhancements. Conversely, when compared against six state-of-the-art swarm intelligence algorithms, the improved BKA demonstrated statistically significant superiority in the majority of cases, as evidenced by consistently low *p*-values (*p* < 0.05). This pattern of results substantiates both the evolutionary compatibility of the proposed enhancements and their practical efficacy in outperforming competing methodologies.

### 4.3. Experiment on Engineering Application

**Figure 9:**
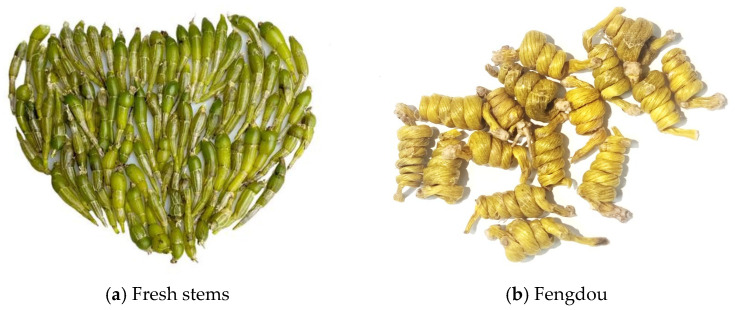
*Dendrobium huoshanense*.

As a highly diverse genus within the Orchidaceae family, *Dendrobium* demonstrates extensive geographical distribution across multiple biogeographical regions. Its primary distribution range encompasses tropical and subtropical Asia, including the Indian subcontinent (India, Nepal, Bhutan, and Sikkim), Southeast Asia (Myanmar, Thailand, Laos, and Vietnam), and substantial habitats throughout China. With global biodiversity estimates reaching approximately 1500 distinct species, *Dendrobium* represents one of the most species-rich genera in the orchid family. Notably, China’s diverse ecosystems harbor about 80 recognized species, accounting for roughly 5.33% of the global *Dendrobium* diversity [39]. Within this diverse genus, *Dendrobium huoshanense*, locally referred to as “Mihu”, represents a critically endangered medicinal species with exclusive endemism to specific montane regions north of the Yangtze River in China. This rare orchid’s highly restricted geographical distribution and ecological specialization contribute to its vulnerable conservation status, emphasizing the need for targeted protection strategies. *Dendrobium* is a traditional Chinese medicine with a wide range of medicinal uses. In 2008, *Dendrobium huoshanense* was proven to be edible by the Ministry of Health and Welfare in Taiwan. In addition, it was listed as the main source of medicinal *Dendrobium* in the 2015 edition of the Pharmacopoeia of the People’s Republic of China, which is shown in Figure 9. *Dendrobium huoshanense* has gained significant scientific and medical attention owing to its remarkable pharmacological properties. Extensive research has demonstrated its multifaceted therapeutic potential, including hepatoprotective effects against acute liver injury, immunomodulatory capabilities, anti-inflammatory benefits in otitis treatment, and promising antineoplastic activities.

Based on established quality grading standards, commercially available *Dendrobium huoshanense* is classified into three distinct categories, namely, super-grade, first-grade, and second-grade products. This classification system facilitates standardized quality assessment and market regulation in the *Dendrobium huoshanense* industry. Since super-grade *Dendrobium huoshanense* has the best medicinal effect and the highest medicinal value, its market price is USD 30,000–50,000 per kilogram, far exceeding that of other grades. If other grades are mixed into super-grade *Dendrobium huoshanense*, it will affect its medicinal effect and even cause medical hazards. In addition, passing off inferior products as superior ones will disrupt the normal trading market of *Dendrobium huoshanense*. Therefore, accurately identifying the grade of *Dendrobium huoshanense* is of vital importance to consumers and relevant market supervision departments.

As there are almost no morphological differences among different grades of *Dendrobium huoshanense*, it is very difficult to visually distinguish the grades of *Dendrobium huoshanense* just from the appearance. Contemporary quality assessment methodologies predominantly rely on analytical techniques, including gas chromatography (GC), high-performance liquid chromatography (HPLC), and DNA-based genetic analysis. While these chemometric approaches provide precise analytical data, they are constrained by multiple operational limitations: complex sample pretreatment protocols, extended analysis periods, substantial reagent consumption, generation of chemical waste streams, and significant procurement and maintenance costs of instrumentation. Furthermore, the technical complexity of these methods hinders their practical implementation by field personnel in production environments. Consequently, there exists a pressing demand to develop and implement innovative analytical platforms that offer rapid, cost-effective, non-destructive, and user-friendly solutions for quality classification and authentication.

This study utilized a comprehensive sample set of 113 *Dendrobium huoshanense* (Fengdou) specimens, categorized into three distinct quality grades based on the standardized classification protocols outlined in DB 34/T 486-2016. The sample distribution comprised 37 super-grade, 37 first-grade, and 39 second-grade specimens. To facilitate robust model development and evaluation, the samples were randomly partitioned into training (*n* = 75) and testing (*n* = 38) subsets using a stratified random sampling approach, ensuring proportional representation of each quality grade in both sets.

**Figure 10:**
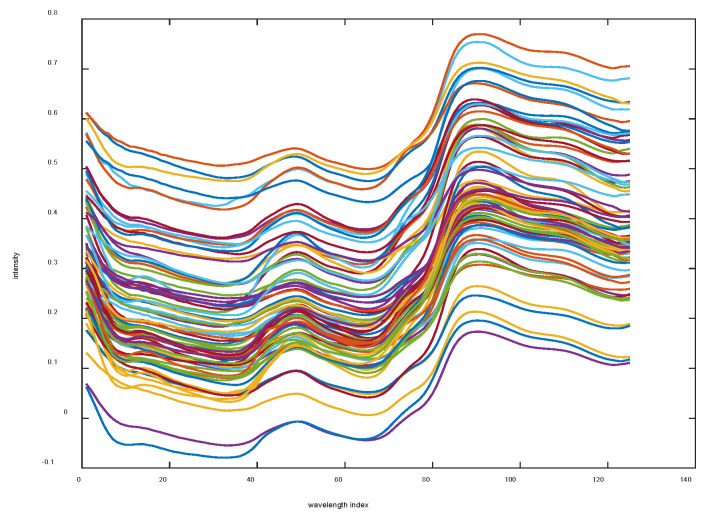
The near-infrared spectral data graph of *Dendrobium huoshanense* (Fengdou).

A support vector machine-based classification model was developed to differentiate between the quality grades. For spectral data acquisition, the samples were analyzed using a microNIRTM 1700 spectrometer equipped with a diffuse reflectance probe. Near-infrared spectra were collected over the full operational wavelength range of the instrument, following established protocols for sample preparation and spectral measurement [40], which is shown in Figure 10. Then, the improved BKA was used to optimize the SVM classification model. The SVM is one of the best performance classifiers, especially the nonlinear classifier with radial basis function (RBF) kernel, which is widely used in a variety of applications. However, as is well known, the SVM classification model based on the RBF kernel function has two important parameters, c and γ. Here, c is the penalty coefficient, which represents the tolerance for errors: a higher c value indicates less tolerance for errors and a higher likelihood of overfitting, while a lower c value makes the model more prone to underfitting. If c is either too large or too small, the generalization ability of the model deteriorates. Additionally, γ implicitly determines the distribution of the data after they are mapped to the new feature space. A larger γ results in fewer support vectors, while a smaller γ leads to more support vectors. Therefore, in order to obtain appropriate values for the parameters c and γ, we applied the improved BKA, BKA, WOA, GOOSE, AO, FOX, HHO, and BWO algorithms to optimize the two parameters of the SVM, respectively, aiming to verify the effectiveness and performance of the improved BKA.

To ensure statistical reliability and minimize experimental randomness, we conducted comprehensive Monte Carlo simulations, performing 30 independent trials for each of the 8 algorithmic approaches in SVM optimization. This rigorous experimental design allowed for robust performance evaluation, with the ensemble results visualized through average convergence trajectories in Figure 9. Quantitative assessment of algorithmic performance is provided in Table 3, which reports the mean recognition accuracy values obtained from testing set evaluations.

The experimental results presented in Table 3 demonstrate the performance superiority of the proposed improved BKA. The comparative analysis revealed that the mean recognition accuracies achieved by the improved BKA, original BKA, WOA, GOOSE, AO, FOX, HHO, and BWO algorithms were 80.66%, 78.95%, 77.5%, 76.97%, 75.66%, 75.92%, 76.71%, and 75.66%, respectively. Statistical analysis indicated that the improved BKA exhibited significant performance enhancement, with accuracy improvements of 1.71%, 3.16%, 3.69%, 5.00%, 4.74%, 3.95%, and 5.00% compared to the benchmark algorithms.

**Figure 11:**
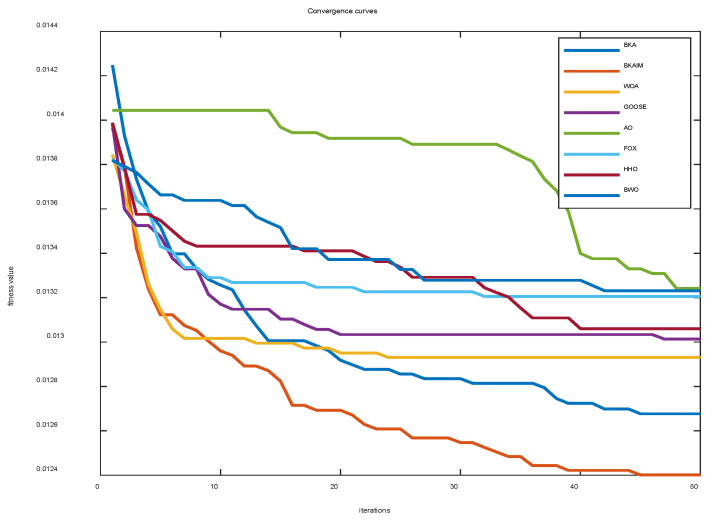
Average convergence curves of the optimized SVM.

Moreover, the improved BKA demonstrated superior stability among the tested algorithms, as evidenced by its minimal standard deviation in recognition accuracy measurements. While all eight algorithms achieved identical maximum recognition accuracy values, the improved BKA distinguished itself through its exceptional convergence speed, as illustrated in Figure 11. This accelerated convergence not only confirmed the enhanced search capability of the proposed algorithm but also underscored its practical engineering applicability in real-world scenarios.

## 5. Conclusions

This study presented an improved black-winged kite optimization algorithm (BKAIM) designed to optimize the search performance of the original BKA framework. The proposed improvement framework incorporated three innovative strategies at distinct algorithm stages.

During population initialization, an opposition-based learning strategy was implemented to create complementary solution pairs, from which an elite subset was selected based on fitness evaluation, effectively enhancing the initial solution quality. In the migration phase, a differential mutation mechanism facilitated inter-population information exchange, simultaneously boosting the global search capability and reducing the premature convergence risk. Furthermore, the conventional absorption boundary method was replaced with a stochastic boundary approach to maintain population diversity, particularly improving solution quality near boundary regions.

The numerical performance of the BKAIM was rigorously evaluated through extensive experimentation on four benchmark test suites: CEC2017, CEC2019, CEC2021, and CEC2022. Comprehensive comparative analysis against the original BKA and six state-of-the-art swarm intelligence algorithms was conducted. Statistical significance, confirmed through the Wilcoxon rank-sum test at a 0.05 significance level, demonstrated that the BKAIM achieved a superior convergence accuracy, faster optimization speed, and enhanced robustness compared to the benchmark algorithms.

To validate the practical utility, the BKAIM was successfully implemented in a real-world application, the identification of *Dendrobium huoshanense* species using near-infrared spectroscopy data combined with SVM classification. The experimental outcomes not only validated the proposed algorithm’s theoretical advantages but also confirmed its reliability and practical applicability in complex, real-world problems.

In the future, a comprehensive research framework for algorithmic enhancement could be established to improve the BKA’s local optima avoidance performance, while simultaneously exploring its potential applications in more complex practical domains. This approach not only addresses the current limitations but also opens new avenues for solving real-world optimization problems using the refined BKA methodology, such as the prediction of polysaccharide content in *Dendrobium huoshanense* and the prediction of coumarin component content in the traditional Chinese medicine of *Peucedanum praeruptorum,* and so on.

## Figures and Tables

**Table 1:** Comparative data of CEC2017 functions’ optimization under eight different algorithms (dim = 10).

Function		BKAIM	BKA	WOA	GOOSE	AO	FOX	HHO	BWO
F1	Best	8.5047 × 10^2^	3.2580 × 10^4^	3.2721 × 10^7^	4.8507 × 10^3^	9.1541 × 10^6^	4.2118 × 10^2^	4.4725 × 10^5^	3.8488 × 10^9^
	Std	7.4898 × 10^8^	1.2300 × 10^9^	1.5191 × 10^8^	1.6964 × 10^9^	1.5442 × 10^8^	1.5123 × 10^9^	2.6048 × 10^7^	5.3647 × 10^9^
	Avg	1.9998 × 10^8^	2.3829 × 10^8^	1.4640 × 10^8^	1.8745 × 10^9^	1.4224 × 10^8^	1.4063 × 10^9^	1.3158 × 10^7^	1.4621 × 10^10^
F3	Best	3.0009 × 10^2^	3.0100 × 10^2^	2.2410 × 10^3^	7.5080 × 10^2^	1.1791 × 10^3^	7.0245 × 10^2^	3.6467 × 10^2^	6.6852 × 10^3^
	Std	1.6156 × 10^3^	3.0713 × 10^3^	1.1127 × 10^4^	1.4804 × 10^4^	1.5438 × 10^3^	3.7196 × 10^3^	8.3279 × 10^2^	2.9780 × 10^3^
	Avg	8.8188 × 10^2^	1.2944 × 10^3^	1.1306 × 10^4^	1.3820 × 10^4^	4.6284 × 10^3^	3.3950 × 10^3^	2.2084 × 10^3^	1.2630 × 10^4^
F4	Best	4.0033 × 10^2^	4.0008 × 10^2^	4.0485 × 10^2^	4.0401 × 10^2^	4.0698 × 10^2^	4.0020 × 10^2^	4.0293 × 10^2^	9.4217 × 10^2^
	Std	2.0195 × 10^1^	3.1380 × 10^1^	6.5805 × 10^1^	1.1838 × 10^2^	1.9343 × 10^1^	1.2817 × 10^2^	3.8479 × 10^1^	6.7983 × 10^2^
	Avg	4.1039 × 10^2^	4.1925 × 10^2^	4.8351 × 10^2^	5.2462 × 10^2^	4.2561 × 10^2^	5.4198 × 10^2^	4.4685 × 10^2^	1.8914 × 10^3^
F5	Best	5.0597 × 10^2^	5.1101 × 10^2^	5.2276 × 10^2^	5.4577 × 10^2^	5.1849 × 10^2^	5.3781 × 10^2^	5.1824 × 10^2^	5.9576 × 10^2^
	Std	1.1833 × 10^1^	1.2963 × 10^1^	2.0303 × 10^1^	2.8085 × 10^1^	1.0102 × 10^1^	2.9144 × 10^1^	2.4412 × 10^1^	1.3536 × 10^1^
	Avg	5.2840 × 10^2^	5.3602 × 10^2^	5.6759 × 10^2^	5.8361 × 10^2^	5.3374 × 10^2^	5.8556 × 10^2^	5.6299 × 10^2^	6.2322 × 10^2^
F6	Best	6.0523 × 10^2^	6.0603 × 10^2^	6.1077 × 10^2^	6.4119 × 10^2^	6.0726 × 10^2^	6.3094 × 10^2^	6.1697 × 10^2^	6.4110 × 10^2^
	Std	5.0381 × 10⁰	7.9189 × 10⁰	1.3752 × 10^1^	1.3303 × 10^1^	8.4823 × 10⁰	1.0133 × 10^1^	1.1560 × 10^1^	7.4097 × 10⁰
	Avg	6.1587 × 10^2^	6.2746 × 10^2^	6.3903 × 10^2^	6.5932 × 10^2^	6.2313 × 10^2^	6.5696 × 10^2^	6.4107 × 10^2^	6.6206 × 10^2^
F7	Best	7.1743 × 10^2^	7.3274 × 10^2^	7.6562 × 10^2^	8.0530 × 10^2^	7.3046 × 10^2^	8.0563 × 10^2^	7.5358 × 10^2^	8.0513 × 10^2^
	Std	1.5992 × 10^1^	1.4459 × 10^1^	1.8286 × 10^1^	1.7579 × 10^2^	1.6132 × 10^1^	7.0224 × 10⁰	1.8240 × 10^1^	1.3876 × 10^1^
	Avg	7.4677 × 10^2^	7.5777 × 10^2^	7.9210 × 10^2^	1.0210 × 10^3^	7.5861 × 10^2^	8.1716 × 10^2^	7.9528 × 10^2^	8.3263 × 10^2^
F8	Best	8.0597 × 10^2^	8.0600 × 10^2^	8.2183 × 10^2^	8.2985 × 10^2^	8.1130 × 10^2^	8.3383 × 10^2^	8.0967 × 10^2^	8.4267 × 10^2^
	Std	8.3534 × 10⁰	9.1981 × 10⁰	1.5162 × 10^1^	2.1949 × 10^1^	7.9554 × 10⁰	1.5566 × 10^1^	1.1040 × 10^1^	7.1202 × 10⁰
	Avg	8.2153 × 10^2^	8.2164 × 10^2^	8.4877 × 10^2^	8.6216 × 10^2^	8.2793 × 10^2^	8.4968 × 10^2^	8.3312 × 10^2^	8.5992 × 10^2^
F9	Best	9.0599 × 10^2^	9.3589 × 10^2^	9.3985 × 10^2^	1.5526 × 10^3^	9.2700 × 10^2^	1.4989 × 10^3^	1.0525 × 10^3^	1.2630 × 10^3^
	Std	9.5970 × 10^1^	1.6041 × 10^2^	3.5577 × 10^2^	5.1820 × 10^2^	1.4632 × 10^2^	7.1298 × 10^1^	2.3244 × 10^2^	2.3550 × 10^2^
	Avg	1.0441 × 10^3^	1.1400 × 10^3^	1.5604 × 10^3^	2.0114 × 10^3^	1.1110 × 10^3^	1.7399 × 10^3^	1.4706 × 10^3^	1.8658 × 10^3^
F10	Best	1.2661 × 10^3^	1.4641 × 10^3^	1.8026 × 10^3^	1.7589 × 10^3^	1.5100 × 10^3^	1.6692 × 10^3^	1.6945 × 10^3^	2.4028 × 10^3^
	Std	2.7799 × 10^2^	1.8277 × 10^2^	2.5617 × 10^2^	3.4515 × 10^2^	2.7854 × 10^2^	3.4032 × 10^2^	2.2175 × 10^2^	1.4932 × 10^2^
	Avg	1.8207 × 10^3^	1.8395 × 10^3^	2.2466 × 10^3^	2.4697 × 10^3^	2.1120 × 10^3^	2.3285 × 10^3^	2.0766 × 10^3^	1.6539 × 10^3^
F11	Best	1.1127 × 10^3^	1.1032 × 10^3^	1.1394 × 10^3^	1.1180 × 10^3^	1.1433 × 10^3^	1.1336 × 10^3^	1.1081 × 10^3^	1.2358 × 10^3^
	Std	5.1406 × 10^1^	3.2965 × 10^1^	9.6683 × 10^1^	1.7548 × 10^3^	3.1673 × 10^2^	2.4641 × 10^3^	8.4895 × 10^1^	9.0255 × 10^3^
	Avg	1.1535 × 10^3^	1.1550 × 10^3^	1.2767 × 10^3^	1.9439 × 10^3^	1.4019 × 10^3^	2.5207 × 10^3^	1.2016 × 10^3^	1.4129 × 10^4^
F12	Best	2.8282 × 10^3^	2.1200 × 10^3^	1.3561 × 10^4^	7.4961 × 10^3^	1.6594 × 10^4^	1.8050 × 10^4^	2.7366 × 10^4^	3.5946 × 10^7^
	Std	1.7019 × 10^5^	1.0131 × 10^5^	5.7040 × 10^6^	2.0869 × 10^6^	4.5933 × 10^6^	2.7235 × 10^6^	5.0532 × 10^6^	4.0763 × 10^8^
	Avg	6.6963 × 10^4^	4.0724 × 10^4^	5.9423 × 10^6^	1.7595 × 10^6^	4.5425 × 10^6^	2.4228 × 10^6^	5.0300 × 10^6^	6.4098 × 10^8^
F13	Best	1.4027 × 10^3^	1.4708 × 10^3^	2.6412 × 10^3^	2.4010 × 10^3^	5.0447 × 10^3^	1.9525 × 10^3^	1.6496 × 10^3^	4.2785 × 10^5^
	Std	4.8410 × 10^2^	3.5566 × 10^3^	1.2962 × 10^4^	1.4569 × 10^4^	1.4387 × 10^4^	1.0908 × 10^4^	1.2486 × 10^4^	4.9530 × 10^7^
	Avg	1.9005 × 10^3^	3.6688 × 10^3^	1.5336 × 10^4^	1.9462 × 10^4^	1.7994 × 10^4^	1.6310 × 10^4^	1.6709 × 10^4^	3.2462 × 10^7^
F14	Best	1.4262 × 10^3^	1.4221 × 10^3^	1.5440 × 10^3^	1.5553 × 10^3^	1.5854 × 10^3^	1.5625 × 10^3^	1.4695 × 10^3^	1.6098 × 10^3^
	Std	2.3930 × 10^1^	3.8163 × 10^1^	2.1474 × 10^3^	7.2346 × 10^3^	1.2847 × 10^3^	7.2755 × 10^3^	1.7012 × 10^3^	1.3609 × 10^3^
	Avg	1.4555 × 10^3^	1.4869 × 10^3^	3.5269 × 10^3^	9.4409 × 10^3^	2.6901 × 10^3^	9.1025 × 10^3^	2.7303 × 10^3^	2.2717 × 10^3^
F15	Best	1.5158 × 10^3^	1.5541 × 10^3^	1.6996 × 10^3^	2.3123 × 10^3^	2.8423 × 10^3^	2.2049 × 10^3^	2.4793 × 10^3^	5.7332 × 10^3^
	Std	4.8849 × 10^1^	1.1281 × 10^2^	1.6056 × 10^4^	5.3159 × 10^4^	6.3406 × 10^3^	3.0200 × 10^4^	5.0525 × 10^3^	1.5759 × 10^3^
	Avg	1.5761 × 10^3^	1.6808 × 10^3^	1.6815 × 10^4^	4.7746 × 10^4^	9.9744 × 10^3^	3.9172 × 10^4^	1.0107 × 10^4^	1.0254 × 10^4^
F16	Best	1.6016 × 10^3^	1.6054 × 10^3^	1.6972 × 10^3^	1.7403 × 10^3^	1.6512 × 10^3^	1.7323 × 10^3^	1.7375 × 10^3^	1.8066 × 10^3^
	Std	1.1340 × 10^2^	1.2520 × 10^2^	1.4470 × 10^2^	2.2768 × 10^2^	1.5754 × 10^2^	1.9669 × 10^2^	1.3533 × 10^2^	1.5274 × 10^2^
	Avg	1.7213 × 10^3^	1.8082 × 10^3^	1.9193 × 10^3^	2.2551 × 10^3^	1.9295 × 10^3^	2.1776 × 10^3^	1.9566 × 10^3^	2.2190 × 10^3^
F17	Best	1.6335 × 10^3^	1.7281 × 10^3^	1.7738 × 10^3^	1.7937 × 10^3^	1.7474 × 10^3^	1.7635 × 10^3^	1.7441 × 10^3^	1.7912 × 10^3^
	Std	2.1180 × 10^1^	2.9817 × 10^1^	7.8686 × 10^1^	1.7231 × 10^2^	2.7053 × 10^1^	1.7871 × 10^2^	6.5043 × 10^1^	5.0915 × 10^1^
	Avg	1.7587 × 10^3^	1.7689 × 10^3^	1.8611 × 10^3^	2.0857 × 10^3^	1.7819 × 10^3^	2.0557 × 10^3^	1.8022 × 10^3^	1.8661 × 10^3^
F18	Best	1.6450 × 10^3^	1.6839 × 10^3^	1.1212 × 10^3^	2.0454 × 10^3^	1.5868 × 10^3^	1.9427 × 10^3^	1.5436 × 10^3^	1.6630 × 10^7^
	Std	4.7913 × 10^3^	4.4599 × 10^3^	1.1039 × 10^4^	1.4725 × 10^4^	5.7316 × 10^4^	1.5825 × 10^4^	8.2694 × 10^3^	5.2919 × 10^8^
	Avg	3.9620 × 10^3^	4.3429 × 10^3^	2.1393 × 10^4^	1.9384 × 10^4^	6.1519 × 10^4^	2.4959 × 10^4^	1.3455 × 10^4^	5.6499 × 10^8^
F19	Best	1.9070 × 10^3^	1.9072 × 10^3^	2.5496 × 10^3^	2.5193 × 10^3^	2.2079 × 10^3^	2.2460 × 10^3^	2.2642 × 10^3^	1.1439 × 10^4^
	Std	1.3689 × 10^2^	2.9430 × 10^1^	1.3846 × 10^6^	1.5248 × 10^4^	9.3702 × 10^4^	2.8529 × 10^4^	4.1560 × 10^4^	3.0598 × 10^6^
	Avg	1.9241 × 10^3^	1.9464 × 10^3^	5.1542 × 10^5^	1.5836 × 10^4^	5.0112 × 10^4^	2.5586 × 10^4^	3.4830 × 10^4^	2.5542 × 10^6^
F20	Best	1.7217 × 10^3^	1.8405 × 10^3^	2.0559 × 10^3^	1.2643 × 10^3^	2.0554 × 10^3^	2.0356 × 10^3^	2.0732 × 10^3^	2.1829 × 10^3^
	Std	2.8340 × 10^1^	6.0864 × 10^1^	7.5746 × 10^1^	1.5827 × 10^2^	6.0274 × 10^1^	1.3223 × 10^2^	9.5625 × 10^1^	4.0949 × 10^1^
	Avg	2.0630 × 10^3^	2.1243 × 10^3^	2.2174 × 10^3^	2.3376 × 10^3^	2.1460 × 10^3^	2.3310 × 10^3^	2.2037 × 10^3^	2.2839 × 10^3^
F21	Best	2.0100 × 10^3^	2.0002 × 10^3^	2.0186 × 10^3^	2.2219 × 10^3^	2.0083 × 10^3^	2.1000 × 10^3^	2.1055 × 10^3^	2.1325 × 10^3^
	Std	6.0253 × 10^1^	6.5619 × 10^1^	4.9773 × 10^1^	4.9480 × 10^1^	4.9355 × 10^1^	8.2335 × 10^1^	5.5157 × 10^1^	6.6866 × 10^1^
	Avg	2.2581 × 10^3^	2.2768 × 10^3^	2.3387 × 10^3^	2.3616 × 10^3^	2.2973 × 10^3^	2.3540 × 10^3^	2.3359 × 10^3^	2.3557 × 10^3^
F22	Best	2.2280 × 10^3^	2.2278 × 10^3^	2.2726 × 10^3^	2.3397 × 10^3^	2.2935 × 10^3^	2.2877 × 10^3^	2.2790 × 10^3^	2.2734 × 10^3^
	Std	4.1388 × 10^1^	1.4150 × 10^2^	4.7118 × 10^2^	6.9312 × 10^2^	1.5225 × 10^1^	5.4399 × 10^2^	1.4356 × 10^1^	4.3866 × 10^2^
	Avg	2.3193 × 10^3^	2.3482 × 10^3^	2.4829 × 10^3^	2.8338 × 10^3^	2.3161 × 10^3^	2.8268 × 10^3^	2.3164 × 10^3^	3.1627 × 10^3^
F23	Best	2.6145 × 10^3^	2.6137 × 10^3^	2.6155 × 10^3^	2.6487 × 10^3^	2.6136 × 10^3^	2.6469 × 10^3^	2.6176 × 10^3^	2.6959 × 10^3^
	Std	1.4646 × 10^1^	2.8849 × 10^1^	3.0630 × 10^1^	5.8074 × 10^1^	1.3923 × 10^1^	5.7828 × 10^1^	3.6208 × 10^1^	3.1085 × 10^1^
	Avg	2.6339 × 10^3^	2.6430 × 10^3^	2.6721 × 10^3^	2.7477 × 10^3^	2.6465 × 10^3^	2.7701 × 10^3^	2.6896 × 10^3^	2.7509 × 10^3^
F24	Best	2.4487 × 10^3^	2.7439 × 10^3^	2.6367 × 10^3^	2.5234 × 10^3^	2.5295 × 10^3^	2.5554 × 10^3^	2.5094 × 10^3^	2.6777 × 10^3^
	Std	8.4675 × 10^1^	3.7398 × 10^1^	4.3052 × 10^1^	1.2378 × 10^2^	6.4195 × 10^1^	8.9719 × 10^1^	8.7859 × 10^1^	1.1052 × 10^2^
	Avg	2.7294 × 10^3^	2.7780 × 10^3^	2.7977 × 10^3^	2.8855 × 10^3^	2.7610 × 10^3^	2.9085 × 10^3^	2.8078 × 10^3^	2.8926 × 10^3^
F25	Best	2.6213 × 10^3^	2.8979 × 10^3^	2.9298 × 10^3^	2.9037 × 10^3^	2.9005 × 10^3^	2.8981 × 10^3^	2.9046 × 10^3^	3.4252 × 10^3^
	Std	6.6058 × 10^1^	6.5928 × 10^1^	4.0212 × 10^1^	7.0507 × 10^1^	2.7587 × 10^1^	4.3195 × 10^1^	2.3445 × 10^1^	2.7518 × 10^2^
	Avg	2.9298 × 10^3^	2.9417 × 10^3^	2.9880 × 10^3^	3.0181 × 10^3^	2.9460 × 10^3^	2.9808 × 10^3^	2.9516 × 10^3^	3.8510 × 10^3^
F26	Best	2.6010 × 10^3^	2.8056 × 10^3^	2.8850 × 10^3^	3.2787 × 10^3^	2.8639 × 10^3^	2.8156 × 10^3^	2.6372 × 10^3^	3.3010 × 10^3^
	Std	2.0657 × 10^2^	4.5311 × 10^2^	5.4822 × 10^2^	4.3862 × 10^2^	1.6137 × 10^2^	7.1953 × 10^2^	5.7104 × 10^2^	3.7349 × 10^2^
	Avg	3.0582 × 10^3^	3.2337 × 10^3^	3.6049 × 10^3^	4.3319 × 10^3^	3.0971 × 10^3^	4.1231 × 10^3^	3.6106 × 10^3^	3.9067 × 10^3^
F27	Best	3.0897 × 10^3^	3.0892 × 10^3^	3.1000 × 10^3^	3.1240 × 10^3^	3.0989 × 10^3^	3.1624 × 10^3^	3.1058 × 10^3^	3.1134 × 10^3^
	Std	1.7868 × 10^1^	2.6097 × 10^1^	4.2266 × 10^1^	5.8417 × 10^1^	5.8198 × 10⁰	7.7883 × 10^1^	6.2653 × 10^1^	5.7165 × 10^1^
	Avg	3.1025 × 10^3^	3.1065 × 10^3^	3.1525 × 10^3^	3.2333 × 10^3^	3.1076 × 10^3^	3.2621 × 10^3^	3.1928 × 10^3^	3.2007 × 10^3^
F28	Best	2.8085 × 10^3^	3.1000 × 10^3^	3.2176 × 10^3^	3.1352 × 10^3^	3.1666 × 10^3^	3.1647 × 10^3^	3.1165 × 10^3^	3.2543 × 10^3^
	Std	1.4359 × 10^2^	1.9187 × 10^2^	1.4439 × 10^2^	1.7817 × 10^2^	1.0934 × 10^2^	1.9829 × 10^2^	1.4300 × 10^2^	1.6502 × 10^2^
	Avg	3.2155 × 10^3^	3.2723 × 10^3^	3.5096 × 10^3^	3.5096 × 10^3^	3.4404 × 10^3^	3.5041 × 10^3^	3.4301 × 10^3^	3.8232 × 10^3^
F29	Best	3.1594 × 10^3^	3.1584 × 10^3^	3.2232 × 10^3^	3.1512 × 10^3^	3.1722 × 10^3^	3.1995 × 10^3^	3.2201 × 10^3^	3.3327 × 10^3^
	Std	3.7381 × 10^1^	6.4538 × 10^1^	1.0879 × 10^2^	1.9343 × 10^2^	7.1430 × 10^1^	2.2561 × 10^2^	9.4104 × 10^1^	8.8604 × 10^1^
	Avg	3.2222 × 10^3^	3.2631 × 10^3^	3.4068 × 10^3^	3.5617 × 10^3^	3.2795 × 10^3^	3.5430 × 10^3^	3.3925 × 10^3^	3.5211 × 10^3^
F30	Best	4.0579 × 10^3^	4.7965 × 10^3^	1.9462 × 10^5^	7.3692 × 10^3^	4.2279 × 10^4^	2.0383 × 10^4^	7.3298 × 10^3^	2.2275 × 10^6^
	Std	4.9682 × 10^5^	1.1250 × 10^6^	2.4932 × 10^6^	7.5555 × 10^6^	2.8637 × 10^6^	1.2953 × 10^7^	2.4151 × 10^6^	7.5472 × 10^6^
	Avg	3.8284 × 10^5^	6.7314 × 10^5^	2.9339 × 10^6^	6.3289 × 10^6^	2.4561 × 10^6^	1.1326 × 10^7^	1.9284 × 10^6^	1.0962 × 10^7^

**Table 2:** Results of the Wilcoxon test.

Function	BKA	WOA	GOOSE	AO	FOX	HHO	BWO
F1	8.2000 × 10^−7^	3.5200 × 10^−7^	1.6400 × 10^−5^	5.1900 × 10^−7^	2.2000 × 10^−7^	1.1100 × 10^−6^	3.0200 × 10^−11^
F3	9.0307 × 10^−4^	4.6200 × 10^−10^	4.6200 × 10^−10^	2.3900 × 10^−8^	1.8500 × 10^−8^	8.3500 × 10^−8^	4.0800 × 10^−11^
F4	9.5207 × 10^−4^	1.8700 × 10^−7^	7.6900 × 10^−8^	7.6000 × 10^−7^	1.7000 × 10^−8^	1.0300 × 10^−6^	3.0200 × 10^−11^
F5	**1.1536 × 10^−1^**	4.5700 × 10^−9^	5.4600 × 10^−9^	**1.2597 × 10^−1^**	1.3300 × 10^−10^	2.0200 × 10^−8^	3.3400 × 10^−11^
F6	1.1900 × 10^−6^	6.7200 × 10^−10^	3.0200 × 10^−11^	9.0307 × 10^−4^	3.0200 × 10^−11^	4.6200 × 10^−10^	3.0200 × 10^−11^
F7	**6.4142 × 10^−1^**	3.5000 × 10^−9^	3.3400 × 10^−11^	5.8737 × 10^−4^	3.0200 × 10^−11^	1.1700 × 10^−9^	3.3400 × 10^−11^
F8	9.4890 × 10^−4^	2.6000 × 10^−8^	1.0900 × 10^−10^	2.8913 × 10^−3^	6.7200 × 10^−10^	7.2000 × 10^−5^	3.3400 × 10^−11^
F9	4.2175 × 10^−4^	2.0300 × 10^−9^	3.0200 × 10^−11^	**9.6263 × 10^−2^**	3.0200 × 10^−11^	1.7800 × 10^−10^	3.0200 × 10^−11^
F10	**5.1060 × 10^−1^**	5.9700 × 10^−5^	1.2000 × 10^−8^	3.6439 × 10^−2^	1.2900 × 10^−6^	1.1143 × 10^−3^	3.0200 × 10^−11^
F11	**1.0869 × 10^−1^**	2.0200 × 10^−8^	4.6200 × 10^−10^	3.8200 × 10^−10^	5.9700 × 10^−9^	1.1738 × 10^−3^	3.0200 × 10^−11^
F12	1.6687 × 10^−2^	2.3700 × 10^−10^	5.5700 × 10^−10^	1.0700 × 10^−9^	1.4100 × 10^−9^	2.3700 × 10^−10^	3.0200 × 10^−11^
F13	9.7900 × 10^−5^	1.0900 × 10^−10^	3.4700 × 10^−10^	6.0700 × 10^−11^	4.2000 × 10^−10^	8.9900 × 10^−11^	3.0200 × 10^−11^
F14	4.4272 × 10^−3^	4.0800 × 10^−11^	4.0800 × 10^−11^	3.0200 × 10^−11^	3.3400 × 10^−11^	6.0700 × 10^−11^	3.0200 × 10^−11^
F15	7.6171 × 10^−3^	3.0200 × 10^−11^	3.0200 × 10^−11^	3.0200 × 10^−11^	3.0200 × 10^−11^	3.0200 × 10^−11^	3.0200 × 10^−11^
F16	4.0127 × 10^−2^	1.0300 × 10^−6^	1.7800 × 10^−10^	3.0059 × 10^−4^	1.6100 × 10^−10^	7.0400 × 10^−7^	4.0800 × 10^−11^
F17	**4.8252 × 10^−1^**	4.4400 × 10^−7^	2.2300 × 10^−9^	4.4592 × 10^−4^	1.7000 × 10^−8^	1.7649 × 10^−2^	4.5000 × 10^−11^
F18	**5.9969 × 10^−1^**	3.5200 × 10^−7^	1.2500 × 10^−7^	5.0700 × 10^−10^	1.1100 × 10^−6^	5.5300 × 10^−8^	3.0200 × 10^−11^
F19	1.3703 × 10^−3^	3.0200 × 10^−11^	3.0200 × 10^−11^	3.0200 × 10^−11^	3.3400 × 10^−11^	3.3400 × 10^−11^	3.0200 × 10^−11^
F20	2.8389 × 10^−4^	1.1000 × 10^−8^	1.6900 × 10^−9^	4.6900 × 10^−8^	1.9600 × 10^−10^	8.1000 × 10^−10^	3.6900 × 10^−11^
F21	4.7460 × 10^−2^	2.7700 × 10^−5^	6.7200 × 10^−10^	1.3703 × 10^−3^	1.0700 × 10^−9^	4.1800 × 10^−9^	8.1500 × 10^−5^
F22	**4.9178 × 10^−1^**	1.1700 × 10^−5^	1.7800 × 10^−10^	2.2800 × 10^−5^	3.2600 × 10^−7^	4.4592 × 10^−4^	8.1500 × 10^−11^
F23	**2.1702 × 10^−1^**	2.2800 × 10^−5^	3.6900 × 10^−11^	3.9881 × 10^−4^	8.9900 × 10^−11^	8.1000 × 10^−10^	3.3400 × 10^−11^
F24	9.4890 × 10^−4^	4.3500 × 10^−5^	4.3100 × 10^−8^	1.3703 × 10^−3^	4.6200 × 10^−10^	2.3800 × 10^−7^	2.8700 × 10^−10^
F25	**6.7350 × 10^−1^**	1.3400 × 10^−5^	5.1900 × 10^−7^	1.5178 × 10^−3^	1.3900 × 10^−6^	**2.4581 × 10^−1^**	3.3400 × 10^−11^
F26	3.3874 × 10^−2^	3.5200 × 10^−7^	2.1500 × 10^−10^	2.7549 × 10^−3^	1.5600 × 10^−8^	6.5200 × 10^−9^	3.3400 × 10^−11^
F27	5.4699 × 10^−3^	4.6400 × 10^−5^	5.5700 × 10^−10^	**1.0233 × 10^−1^**	4.9800 × 10^−11^	3.3500 × 10^−8^	5.0000 × 10^−9^
F28	**9.1171 × 10^−1^**	7.2200 × 10^−6^	2.8300 × 10^−8^	1.3100 × 10^−8^	1.1600 × 10^−7^	7.2200 × 10^−6^	3.0200 × 10^−11^
F29	1.9527 × 10^−3^	7.3800 × 10^−10^	8.9900 × 10^−11^	2.1300 × 10^−5^	4.6200 × 10^−10^	5.0000 × 10^−9^	3.0200 × 10^−11^
F30	2.2360 × 10^−2^	1.8700 × 10^−7^	1.8600 × 10^−9^	3.9600 × 10^−8^	1.4300 × 10^−8^	5.5300 × 10^−8^	3.6900 × 10^−11^

Values with *p* > 0.05 are bolded to indicate insignificance.

**Table 3:** The recognition results of eight methods on the test set.

Methods	BKAIM	BKA	WOA	GOOSE	AO	FOX	HHO	BWO
Best	81.58	81.58	81.58	81.58	81.58	81.58	81.58	81.58
Std	1.29	2.26	3.57	3.18	3.29	3.94	3.34	2.54
Avg	80.66	78.95	77.5	76.97	75.66	75.92	76.71	75.66

## Data Availability

The datasets and source code are available upon reasonable request to the corresponding author.
